# Inhibitory Effect of 2,3,5,6-Tetrafluoro-4-[4-(aryl)-1*H*-1,2,3-triazol-1-yl]benzenesulfonamide Derivatives on HIV Reverse Transcriptase Associated RNase H Activities

**DOI:** 10.3390/ijms17081371

**Published:** 2016-08-20

**Authors:** Nicolino Pala, Francesca Esposito, Dominga Rogolino, Mauro Carcelli, Vanna Sanna, Michele Palomba, Lieve Naesens, Angela Corona, Nicole Grandi, Enzo Tramontano, Mario Sechi

**Affiliations:** 1 Dipartimento di Chimica e Farmacia, Università di Sassari, Via Vienna 2, I-07100 Sassari, Italy; vsanna@uniss.it (V.S.); mpalomba@uniss.it (M.P.); 2 Dipartimento di Scienze della Vita e dell’Ambiente-Sezione Biomedica, Università di Cagliari, Cittadella Universitaria SS554, I-09042 Monserrato, Italy; francescaesposito@unica.it (F.E.); angela.corona@unica.it (A.C.); nicole.grandi2@gmail.com (N.G.); tramon@unica.it (E.T.); 3 Dipartimento di Chimica, Università di Parma, Parco Area delle Scienze 17/A, I-43124 Parma, Italy; dominga.rogolino@unipr.it (D.R.); mauro.carcelli@unipr.it (M.C.); 4 Rega Institute for Medical Research, KU Leuven, B-3000 Leuven, Belgium; lieve.naesens@kuleuven.be; 5 Istituto di Ricerca Genetica e Biomedica, Consiglio Nazionale delle Ricerche (CNR), I-09042 Monserrato, Italy

**Keywords:** HIV-1 RNase H, click-chemistry, 4-[4-(aryl)-1*H*-1,2,3-triazol-1-yl]benzenesulfonamides, docking

## Abstract

The HIV-1 ribonuclease H (RNase H) function of the reverse transcriptase (RT) enzyme catalyzes the selective hydrolysis of the RNA strand of the RNA:DNA heteroduplex replication intermediate, and represents a suitable target for drug development. A particularly attractive approach is constituted by the interference with the RNase H metal-dependent catalytic activity, which resides in the active site located at the C-terminus p66 subunit of RT. Herein, we report results of an in-house screening campaign that allowed us to identify 4-[4-(aryl)-1*H*-1,2,3-triazol-1-yl]benzenesulfonamides, prepared by the “click chemistry” approach, as novel potential HIV-1 RNase H inhibitors. Three compounds (**9d**, **10c**, and **10d**) demonstrated a selective inhibitory activity against the HIV-1 RNase H enzyme at micromolar concentrations. Drug-likeness, predicted by the calculation of a panel of physicochemical and ADME properties, putative binding modes for the active compounds, assessed by computational molecular docking, as well as a mechanistic hypothesis for this novel chemotype are reported.

## 1. Introduction

Infections with Human Immunodeficiency Virus Type-1 (HIV-1), the causative agent of Acquired Immunodeficiency Syndrome (AIDS), constitute a serious and global health problem [1,2,3]. Due to the lack of an effective vaccine, antiretroviral treatment remains the only option for control, allowing HIV-1 infection to transform from a highly lethal syndrome into a chronic condition. However, the use and long-term effectiveness of these drugs have severe limitations, in particular the intrinsic drug toxicity during long-term administration, the emergence of drug-resistant strains, poor patient compliance necessitating careful monitoring, and considerable costs. The current approach (also termed High Active Anti-Retroviral Therapy, HAART) involves the combined inhibition of two or three viral enzymes, protease (PR), integrase (IN), and especially reverse transcriptase (RT), which are all essential for the virus to replicate.

Besides these validated pharmacological targets, the inhibition of the ribonuclease H (RNase H) catalytic activity of the multifunctional RT enzyme has not yet been extensively explored as a suitable antiviral strategy [4,5,6,7,8].

The HIV RT enzyme is associated with RNA- and DNA-dependent polymerase, RNase H strand displacement, and strand transfer activities, which are required to transcribe the single-stranded vRNA into double-stranded proviral vDNA.

**Figure 1:**
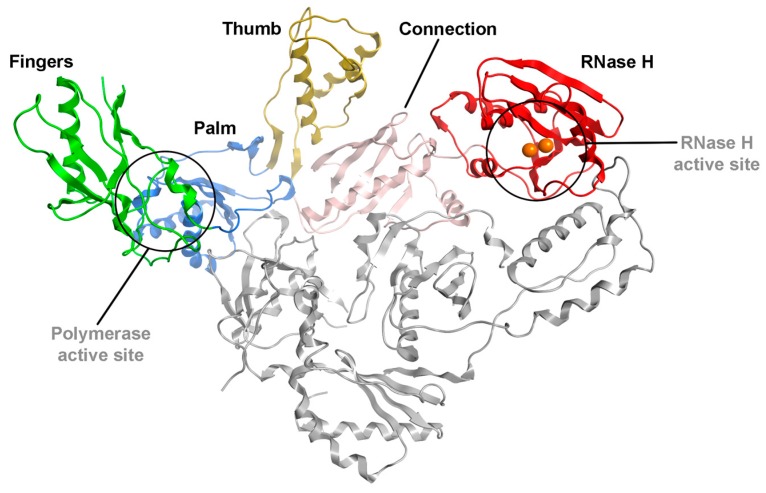
Overall view of the full HIV-1 RT heterodimer. Subunits p51 (grey) and p66 (which include fingers, palm, thumb, connection, and RNase H sites) are depicted as ribbon. Polymerase and RNase H active sites are circled in black. The RNase H metal cofactors are represented as orange spheres.

From the structural point of view, RT is an asymmetrical heterodimer originating from two copies of the p66 polypeptide that is encoded by the HIV pol gene. During viral maturation the C-terminal portion of one p66 subunit is shortened by about 15 kDa, thus yielding the p51 polypeptide, which is assembled with an uncut copy of p66 to form the mature enzyme. All the enzymatic activities reside on the p66 subunit, whereas p51 lacks catalytic functions and only acts as a structural support. Subunit p66 can be divided into five distinct domains; three of these are organized in a thumb-palm-fingers fashion, and are the depository of the polymerase activities. A fourth connection domain separates the hand from the RNase H domain located at the C-terminus of p66 (Figure 1).

At present, more than 30 HIV blockers have been approved, with RT being the most successful viral target. All marketed RT inhibitors belong to the classes of nucleoside/nucleotide RT inhibitors (N(t)RTIs) or non-nucleoside RT inhibitors (NNRTIs), and act by interfering with the polymerase activities of RT. However, despite the relatively high number of available compounds for combination therapy, drug effectiveness is hampered by the rapid emergence of drug-resistant viruses. One possible way to overcome this problem would be to inhibit the RNase H activity of RT. In fact, it is highly plausible that an additional RT inhibition mechanism could contribute synergistically to supress viral replication.

From the functional point of view, HIV-1 RNase H, which catalyzes hydrolysis of the RNA part of the replicative intermediate DNA:RNA hybrid, is a member of the polynucleotidyl transferase superfamily. The tertiary structure of the HIV-1 RT RNase H active site is similar to that of all known RNases H, and contains five highly conserved residues that are essential for the catalysis: Asp443, Glu478, Asp498, Asp549 (the “DEDD motif”), and His539 [5,8,9]. Moreover, high resolution crystallography of HIV-1 RT RNase H complexed to a RNA/DNA hybrid revealed that the DEDD motif stabilizes two Mg^2+^ ions, which promotes cleavage of the phosphodiester bond of the RNA chain, according to the “two-metal ions mechanism” [10,11].

**Figure 2:**
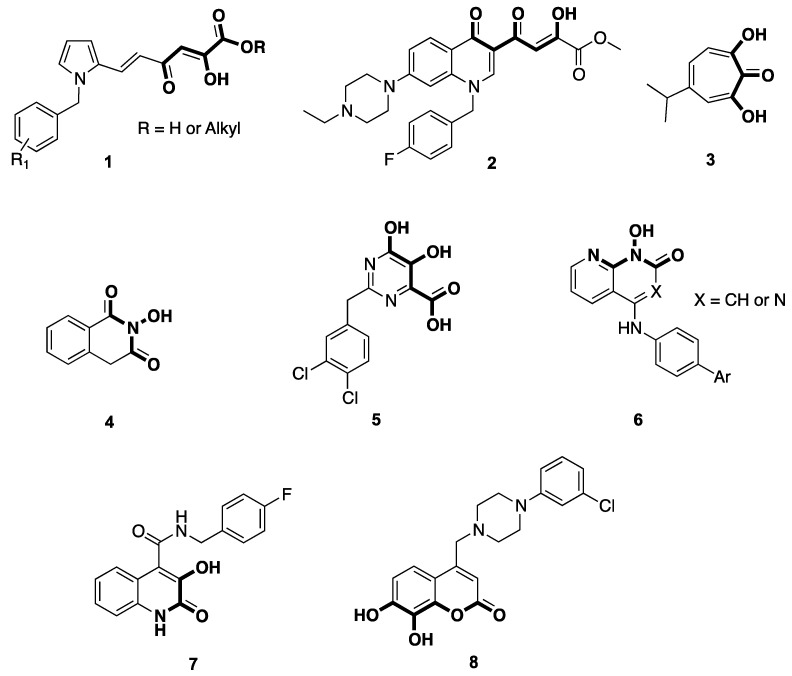
Structures of some representative HIV-1 RNase H inhibitors.

As demonstrated for other polynucleotidyl transferases, HIV-1 RNase H can also be inhibited through selective chelation of the metal cofactors [12]. During the last years, a few classes of compounds capable of inhibiting HIV-1 RNase H activity by involving such a mechanism have been identified (Figure 2), and include diketoacids/esters (**1**) [13,14,15,16], triketoacids (**2**) [17], β-thujaplicinol (**3**) [18], 2-hydroxyisoquinoline-1,3(2*H*,4*H*)-diones (HID, **4**) [19], pymidinol carboxylic acids (**5**) [20], naphthyridinones (**5,6**) [21,22], 3-hydroxy-2-oxo-1,2-dihydroquinoline-4-carboxamides (**7**) [23], and 4-(aryl)piperazin-1-yl)methyl)-7,8-dihydroxy-2*H*-chromen-2-ones (**8**) [24]. All these compounds share a common pharmacophoric pattern constituted by at least three metal chelating features, which usually consist of three oxygen atoms (compounds **1**–**5**, and **8**). In other examples, as outlined by compounds **6** and **7**, the minimal pharmacophore is formed by the combination of two oxygen atoms and one nitrogen atom. However, neither of these compounds is currently moving into advanced preclinical development.

**Figure 3:**
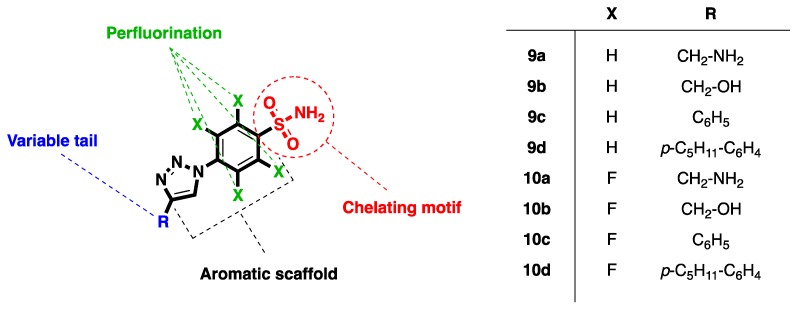
General structure of compounds **9a**–**d** and **10a**–**d**.

In the course of a focused screening campaign using an in-house collection of molecules specifically designed as metalloenzyme inhibitors, we found that compounds bearing the 4-(aryl–triazolyl)-benzenesulfonamide scaffold can act as novel HIV-1 RNase H inhibitors. Herein, we report results on the HIV-1 RNase H, RT-associated DNA polymerase (DP) and HIV-1 integrase (IN) inhibitory activities of two sets of 4-[4-(substituted)-1*H*-1,2,3-triazol-1-yl]benzenesulfonamide derivatives (**9a**–**d** and **10a**–**d**, Figure 3), which we selected to also evaluate the influence of the perfluorination on the benzenesulfonamide chemotype. Moreover, we present a typical panel of physicochemical ADME parameters for the most active compounds, the interaction between a model ligand and the Mg^2+^ ions, as well as computational docking studies to predict the possible binding mode of our compounds within the enzyme active site.

## 2. Results and Discussion

### 2.1. Compound Identification

The possibility that some metal-chelating inhibitors can block not only the specific target for which they were designed, but also other metalloenzymes, has proven to be a consolidated strategy for medicinal chemists [24]. In particular, this approach led us to identify novel classes of influenza virus endonuclease inhibitors (i.e., salicyl thiosemicarbazones [25], and acyl hydrazones [26]), as well as original prototypes (i.e., pyrazole-carboxylic acids) of carbonic anhydrase inhibitors (CAIs) [27]. On this basis, we evaluated some representative compounds belonging to a general class of 4-(4-substituted-triazole)-benzenesulfonamides, specifically designed as CAIs, as putative HIV-1 RT-associated RNase H inhibitors.

To start, a library of selected metal-chelating compounds was preliminarily screened in order to assess their ability to inhibit the HIV-1 RNase H activity. First, from an in-house library of about 250 compounds, we chose structural backbones carrying metal-coordinating functionalities. Next, after a preliminary screening against the HIV-1 RNase H enzyme, a few hit compounds belonging to a novel class of 4-(4-substituted-triazole)-benzenesulfonamides were identified. Among them, our attention was drawn to derivatives **9a**–**d** and **10a**–**d** (Figure 3), which were submitted for further investigation. Although the interaction between sulfonamides and Zn^2+^ ions is widely investigated and established, coordination with Mg^2+^ appeared relatively novel; however, it is well known that deprotonated oxygens are likely to establish an interaction with hard Lewis acid-like ions such as Mg(II). It is worth nothing that the selected compounds **9a**–**d** and **10a**–**d** are representative of a collection previously prepared by us using copper-catalyzed azide-alkyne cycloaddition (CuAAC) [28].

CuAAC is one of the most successful click chemistry methods because it allows us to create large libraries of complex compounds with chemical diversity, starting from simple reactants [29,30]. Moreover, CuAAC reactions are usually high-yielding under facile and mild conditions, with the possibility to generate and manage stereoselectivity.

In this scenario, compounds **9a**–**d** and **10a**–**d** were selected to preliminarily explore this chemical space, in order to achieve a certain chemical diversity by modulating both the steric hindrance and hydrophilic/lipophilic balance. Namely, the nature of the R moiety on the triazole was planned considering various chemical functionalities including alkyl-(substituted)aromatic, hydroxyalkyl- or aminoalkyl-substituents (Figure 3).

On the other hand, complete substitution of the hydrogens of an aromatic ring with fluorine atoms is a well-known bioisosteric replacement strategy that, among other effects, dramatically increases the acidity of ring substituents without significantly affecting the steric properties of the molecule [31]. In fact, the atomic size of fluorine is comparable to that of hydrogen, but its high electronegativity produces several changes such as decrease of pK_a_, the modulation of lipophilicity and other physicochemical parameters, which can lead to substantial variation of physicochemical properties [31].

In order to study the influence of perfluorination on the activity of homologous compounds, we re-synthesized two subclasses of derivatives (Scheme S1), where the first bears a simple benzenesulfonamide scaffold (**9a**–**d**), while the second one carries a 2,3,5,6-tetrafluorobenzenesulfonamide group (**10a**–**d**).

Moreover, we sought to predict the pK_a_ of the sulfonamide group of these two sets of compounds, under the presence or absence of perfluorination on the benzenesulfonamide ring. From a computational prediction [32], the pK_a_s were lowered approximately from six to 3.5, for compounds **9a**–**d** and **10a**–**d**, respectively, and this should dramatically affect the ion speciation profile of the tested compounds. In particular, at pH 7.4 the sulfonamide functionality of compounds **9a**–**d** would prevalently exist in non-deprotonated form, while at the same pH deprotonation of the sulfamoyl group on fluorinated **10a**–**d** should be favored. It was also supposed that the presence of a negatively charged sulfonamide could increase the affinity of these compounds for the metal cofactors of HIV-1 RNase H.

### 2.2. Interaction with Mg^2+^

**Figure 4:**
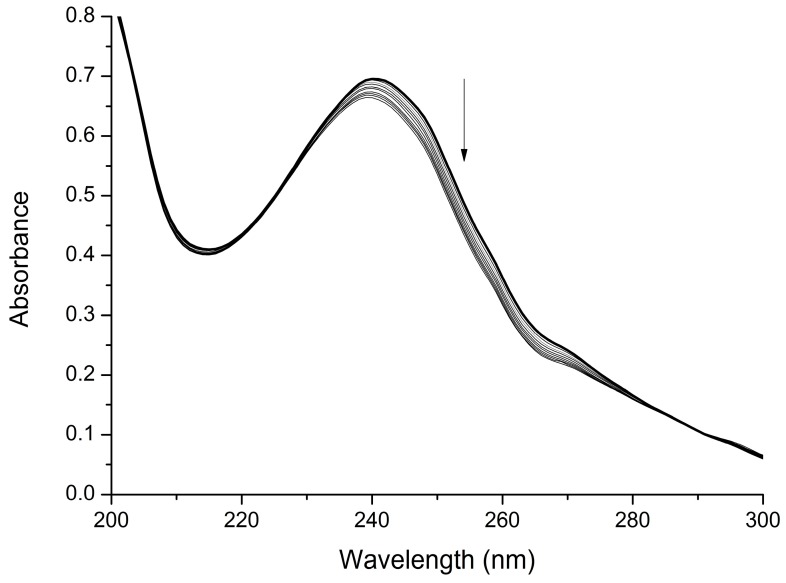
UV-vis titration of ligand **10d** (bold line, compound alone) with increasing amount of Mg(CH_3_COO)_2_. The arrow indicates the direction of absorbance change as Mg(CH_3_COO)_2_ increases.

Since RNase H active site inhibitors have been reported to chelate the Mg^2+^ ions [12,13,14,15,16,17,18,19,20,21,22,23,24], and to support the hypothesis that the sulfonamide group could interact with the metal cofactors in the RNase H catalytic pocket, we measured the capability of the model compound **10d** to coordinate Mg^2+^ ions by means of UV-visible (UV-vis) experiments. A series of UV-vis absorbance spectra of a methanolic solution of **10d** in the presence of increasing equivalents of Mg(CH_3_COO)_2_ were recorded (Figure 4). We observed that increasing concentrations of Mg^2+^ produced a reduction of the maximum UV-vis absorption, supporting the involvement of coordinative interactions between the sulfonamide and metal ions.

### 2.3. Biology

An enzymatic assay using recombinant HIV-1 RT and IN proteins was performed to assess the ability to inhibit HIV-1 RT-associated RNase H and DP functions, and/or the IN catalytic process. Within the series of **9a**–**d** and **10a**–**d**, three compounds (**9d**, **10c**, and **10d**) were endowed with selective inhibitory activity towards RNase H (Table 1).

With an IC_50_ value of 6.6 ± 0.5 µM, **10d** was the most active compound of the series. Marginal or moderate activities were registered for **9d** (IC_50_ = 63 ± 7 µM) and **10c** (IC_50_ = 26 ± 3 µM), respectively. However, **10d** and **10c** also exhibited inhibition of the DP function (IC_50_ 33.4 ± 5.8 and 90 ± 5 µM, for **10d** and **10c**, respectively, Table 1), meaning that the potency for DP inhibition was much lower compared to that for RNase H inhibition. In fact, their specificity indexes (SpI, expressed as the ratio of the RNase H IC_50_ value vs. the DP IC_50_ value), were favorable to the RNase H function (SpI = ~5 and ~3.5, for **10d** and **10c**, respectively). Moreover, these compounds were inactive when tested for IN inhibition (in the presence of the LEDGF cofactor and with 100 µM as the highest test concentration). Overall, these data would support a relative selectivity toward RNase H enzymatic activity.

Although the number of tested compounds was not sufficient to perform a detailed structure-activity relationships (SAR) analysis, these results allowed us to identify two key features that seem important for activity: (a) the biaryl-triazole moiety, free or with an appropriate substituent (i.e., a *n*-pentyl tail); and (b) the perfluorination on the benzenesulfonamide ring. For example, since two of the three active compounds possess both these features, we can hypothesize that the simultaneous presence of the biaryl-triazole moiety and a small aliphatic chain in the position para may be a crucial role for the activity. This can also be supported by the observation that small polar substituents (such as OH or NH_2_) in position 4 of the triazole ring, as in compounds **9a**,**b**, and **10a**,**b**, dramatically abolish the activity. These findings are consistent with those reported by Williams [21], Gerondelis [33], and Vernekar [34], who suggested that the presence of an accessory biaryl motif, in addition to the aromatic scaffold bearing the chelating group, is an important factor for HIV-1 RNase H inhibition.

As far as the perfluorination is concerned, the presence of tetrafluorine on the benzenesulfonamide group led to an increase in potency, as observed by comparing the IC_50_ values of **9c** and **10c** (from >100 µM to 26 µM), and **9d** and **10d** (from 63 µM to 6.6 µM), respectively. 

Finally, the presence of the *n*-pentyl tail on the biaryltriazole shares an additional positive effect, as observed by comparing the activity of compounds **9c** and **9d** (IC_50_ > 100 µM and 63 µM, respectively), and of **10c** and **10d** (IC_50_ 26 µM and 6.6 µM, respectively).

### 2.4. Absorption, Distribution, Metabolism and Excretion Prediction

Determination of physicochemical properties and the related absorption, distribution, metabolism and excretion (ADME) properties is a useful approach to predict the druggability and therapeutic potential of a lead candidate [35]. Therefore, calculation of a panel of selective parameters important to predict the solubility and membrane permeability for the most interesting compounds **9d**, **10c**, and **10d** was performed by considering Lipinski’s rule-of-five method (Table 2). According to this procedure, a compound is likely to be well absorbed when it possesses a molecular weight <500, number of H-bond acceptors <10, number of H-bond donors <5, and log P < 5 [36]. Moreover, other parameters, such as the log S (−6.5 to 0.5) and the topological polar surface area (TPSA < 140 Å^2^), that correlate with membrane permeability have been considered [37,38,39]. As shown in Table 2, the calculated atom-based values (i.e., molecular weight, H-bond acceptor and donor counts, log P, log S and TPSA) for **9d**, **10c**, and **10d** meet desirable ADME criteria for good absorption and membrane permeability, and are likely to yield favorable pharmacokinetic properties and bioavailability.

### 2.5. Docking

**Figure 5:**
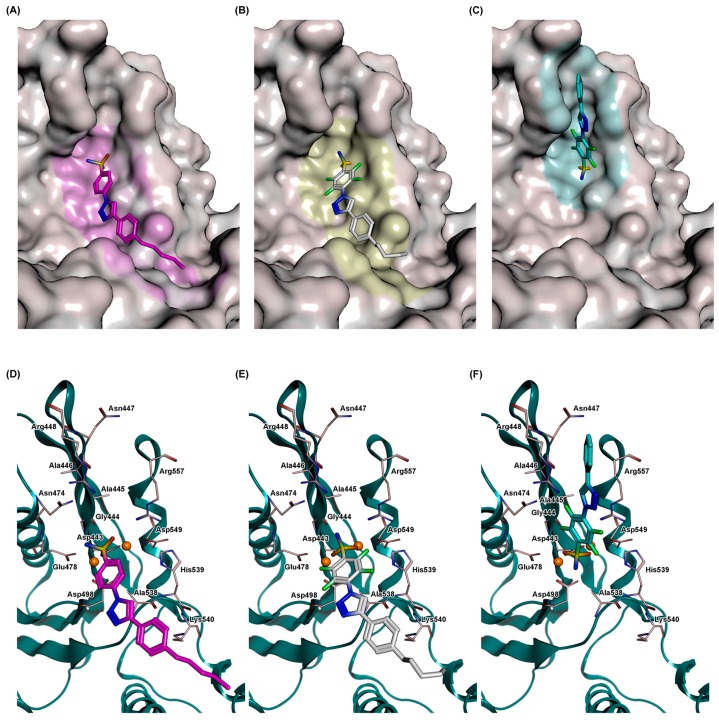
Predicted binding modes for the most active compounds **9d**, **10c**, and **10d** within the HIV-1 RNase H catalytic site: (**A**–**C**) best docking pose for **9d** (purple), **10c** (cyan), and **10d** (white), respectively, with the protein shown as grey surface, and with the contact residues colored in the same color as the docked ligand; (**D**–**F**) close views of the binding site, where the protein is depicted as cartoon (blue lagoon), side chains of relevant residues are provided as thick line (pink), and Mn^2+^ metal cofactors as spheres (orange).

To predict the putative binding mode of the selected compounds **9d**, **10c**, and **10d**, a series of computational docking studies were performed. As displayed in Figure 5, docking results for the three active compounds (**9d**, **10c**, and **10d**) revealed a tight affinity within the amino acid pocket located near the catalytic site, which includes the following residues: Asp443-Arg448, Asn474, Glu478, Asp498, Ala538-Lys540, and Asp549 (Figure 5, Figure S1). Although the common sulfonamide function of the selected compounds is directed toward the metal ions, the orientation of their aromatic backbones appears different within two pockets, probably depending on the presence (**9d** and **10d**) or absence (**10c**) of the aliphatic *n*-pentyl tail. In particular, the most favorable conformation of **9d** and **10d** is placed along the surface of the protein, with the 4-pentylphenyltriazole accommodated in a cavity surrounding the active site formed by residues Asn265, Trp266, Ser268, Gln269, Ile274, and Lys275 (Figure 5A,C for **9d** and **10d**, respectively). Strong arene-cation interactions between the 4-pentylbenzene ring and Lys540, for both **9d** and **10d**, were revealed. Otherwise, compound **10c** (Figure 5B) is engaged into a second pocket, with the benzene ring placed in a narrow cavity lined by residues Ala446-Glu449, Ile556, and Arg557, in close proximity to the catalytic site. Similarly to **9d** and **10d**, the distal benzene ring of **10c** is also involved in an arene-cation interaction, in this case with Arg448, which establishes a second arene-cation interaction with the triazole ring.

Significant differences could be rationalized on the coordination of metal cofactors by each compound. In particular, in **9d** (dG = 11.9648 kcal·mol^−1^, IC_50_ = 63 µM), which bears a neutral sulfonamide group, one oxygen of the sulfonamide group is bridging between the two metal cofactors (Mn^2+^ ions are present in the 3LP2 X-ray structure, Figure S1A). Comparatively, each oxygen of the sulfonamide group of **10c** (dG = 14.2330 kcal·mol^−1^, IC_50_ = 26 µM) appeared involved in one bond (two bonds in total) with one metal ion (Figure S1B): in this way, the ligand produced µ_2_-bridging between the two metal ions. Interestingly, the most active derivative **10d** (dG = 19.4250 kcal·mol^−1^, IC_50_ = 6.6 µM) is predicted to coordinate one manganese cation with both sulfonamide oxygens; one of the two chelating oxygens shows an additional interaction with the second cation (Figure S1C). These interactions would presumably concur, together with the molecules of the solvent and with amino acids on the catalytic pocket, to complete the coordination geometry around the metal, favoring chelation of metal cofactors and, consequently, the inhibitory activity.

These results suggest that the activity of these compounds can be explained by their arrangement in the active site of the enzyme. The superior potency of **10d** (IC_50_ = 6.6 ± 0.5 µM) can be attributed to the optimal pK_a_ of the sulfonamide group, as well as to a favorable orientation of the chelating motif, which should significantly contribute to its metal coordination.

## 3. Materials and Methods

### 3.1. Chemistry

Compounds **9a**–**d** and **10a**–**d**, were prepared following a synthetic procedure previously reported by us [28]. The experimental details and characterization data are detailed in the Supporting Information. Briefly, the title compounds **9a**–**d** and **10a**–**d** were synthesized by reacting the azides **11** and **12** with alkynes **13b**–**d**,**e** in the presence of nanosized metallic copper as catalyst (Scheme S1) [28].

### 3.2. UV-Visible Titration

UV-vis absorption spectra of **10d** were registered by a spectrophotometer uniSPEC 2 (LLG Labware, BDL Czech Republic sro, Turnov, Czech Republic) using a 0.025 mM solution in methanol. Each metal/ligand (M:L) system was studied by titrating a 3.0 mL of the ligand solution with a methanolic solution of Mg(CH_3_COO)_2_ (5 mM); spectra of samples with M:L molar ratio ranging from 0 to 10 were measured.

### 3.3. Biology

#### 3.3.1. Protein Expression and Purification

The recombinant HIV-1 RT protein, the coding gene of which was subcloned in the p6HRT_prot plasmid, was expressed in *E. coli* strain M15 [40,41]. The bacteria cells were grown up to an optical density (at 600 nm) of 0.8 and induced with 1.7 mM isopropyl β-d-1-thio galactopyranoside (IPTG) for 5 h. HIV-1 RT purification was performed as described [42]. Briefly, cell pellets were re-suspended in lysis buffer (20 mM HEPES, pH 7.5; 0.5 M NaCl; 5 mM β-mercaptoethanol; 5 mM imidazole; 0.4 mg·mL^−1^ lysozyme), incubated on ice for 20 min, sonicated, and centrifuged at 30,000× *g* for 1 h. The supernatant was applied to a His-binding resin column and washed thoroughly with wash buffer (20 mM HEPES, pH 7.5; 0.3 M NaCl; 5 mM β-mercaptoethanol; 60 mM imidazole; 10% glycerol). RT was eluted by imidazole gradient, and the enzyme-containing fractions were pooled and dialyzed and aliquots were stored at −80 °C.

#### 3.3.2. HIV-1 RNase H Polymerase-Independent Cleavage Assay

The HIV-1 RT-associated RNase H activity was measured as described [42] in 100 μL reaction volume containing 50 mM Tris HCl, pH 7.8; 6 mM MgCl_2_, 1 mM dithiothreitol (DTT), 80 mM KCl, 0.25 µM hybrid RNA/DNA (5′-GTT TTC TTT TCC CCC CTG AC-3′-fluorescein, 5′-CAA AAG AAA AGG GGG GAC UG-3′-dabcyl) and 3.8 nM RT. The reaction mixture was incubated for 1 h at 37 °C. The enzymatic reaction was stopped with the addition of ethylenediaminetetraacetic acid (EDTA) and measured with a Victor3 instrument (Perkin) at 490/528 nm.

#### 3.3.3. HIV-1 RT-Associated RNA-Dependent DNA Polymerase Activity Determination

The HIV-1 RT-associated RNA-dependent DP activity was measured as previously described [23]. Briefly, 20 ng of HIV-1 wt RT was incubated for 30 min at 37 °C in 25 mL volume containing 60 mM Tris HCl, pH 8.1, 8 mM MgCl_2_, 60 mM KCl, 13 mM DTT, 2.5 mM poly(A)-oligo(dT), 100 mM dTTP. Enzymatic reaction was stopped by addition of EDTA. Reaction products were detected by picogreen addition and measured with a PerkinElmer Victor 3 multilabel counter plate reader at excitation-emission wavelength of 502/523 nm. Chemical reagents were purchased form Sigma Aldrich srl. RNA-DNA labelled sequences were purchased from Metabion international AG.

#### 3.3.4. HIV-1 IN/LEDGF HTRF LEDGF-Dependent Assay

Recombinant IN and LEDGF/p75 were purified as described by Esposito et al. [43]. The INLEDGF/p75-dependent assay allow to measure the inhibition of 3′-processing and strand transfer IN reactions in presence of recombinant LEDGF/p75 protein, as previously described [44]. Briefly, 50 nM IN was pre-incubated with increasing concentration of compounds for 1 h at room temperature in reaction buffer containing 20 mM HEPES pH 7.5, 1 mM DTT, 1% Glycerol, 20 mM MgCl_2_, 0.05% Brij-35 and 0.1 mg/mL BSA. DNA donor substrate, DNA acceptor substrate and 50 nM LEDGF/p75 protein were added and incubated at 37 °C for 90 min. After the incubation, 4 nM of Europium-Streptavidine were added at the reaction mixture and the HTRF signal was recorded using a Perkin Elmer Victor 3 plate reader using a 314 nm for excitation wavelength and 668 and 620 nm for the wavelength of the acceptor and the donor substrates emission, respectively.

### 3.4. Molecular Modeling

#### 3.4.1. Hardware Specifications

All calculations were performed on a 64 bit Intel 8-Core i7-2600 CPU (Hewlett Packard, Palo Alto, CA, USA) running at 3.40 GHz with 8 GB RAM.

#### 3.4.2. Protein Preparation

The coordinates of full-length mutant HIV-1 RT were retrieved from RCSB Protein Data Bank (accession code 3LP2). Wild-type enzyme was obtained by retro-mutation of Asp103 to Lysine, then the missed residue Arg557 belongings to the HIV-1 RNase H active site was modeled using the crystal complex 3K2P, as previously described [14]. The protein was prepared using Molecular Operating Environment software package platform (MOE, version 2009.10, Chemical Computing Group Inc., Montreal, QC, Canada) [45] as follows: solvent molecules were removed, and chains termini were capped; then all hydrogens were added to the system, partial atomic charges were assigned according OPLS_AA force field, and minimization procedure was applied in order to optimize atoms positions.

#### 3.4.3. Ligands Preparation

The ligands were built using MOE builder mask. For each ligand the predicted most representative species at pH 7.4 was considered. Thus, compounds **9c** was modeled as neutral species, whereas for compounds **10c** and **10d**, due to the tetrafluorination, the mono-deprotonated sulfonamide form was considered. The geometries of the ligands were optimized by an energy minimization pass until a convergence gradient of 0.01 kJ (mol·Å)^−1^ was reached using the MMFF94x force field. Solvent effect was calculated using the Generalized Born Solvation Model.

#### 3.4.4. Docking Procedures

Triangle Matcher Placement docking method implemented in MOE platform was used to re-dock the co-crystallized ligand of 3LP1 on the HIV-1 RNase H active site. The results were scored using London dG as rescoring, Forcefield as refinement, and Affinity dG as second rescoring functions. The same protocol was applied to the database containing our ligands.

## 4. Conclusions

In summary, we identified original 4-[4-(substituted)-1*H*-1,2,3-triazol-1-yl]benzenesulfonamides as potential inhibitors of the HIV-1 RNase H function. This study highlighted that key features such as a (substituted) biaryl moiety and perfluorination on the benzenesulfonamide ring are crucial for the inhibitory activity of this class of compounds. These insights resulted in compound **10d** as a representative and interesting lead compound for drug optimization. Moreover, spectroscopic analyses and molecular modeling studies indicated that such derivatives can mechanistically act by interfering with the two-metal ions through direct coordination of the metal cofactors in the enzyme catalytic site. This is in agreement with the behavior of diketo/triketoacid derivatives [14], but differs from what was observed for allosteric inhibitors [6,18] and dual inhibitors of RNase H and RT-associated DP activity, which were recently proposed to bind to a site close, but not coincident, to the RNase H active site [46]. Finally, calculated ADME properties and the preliminary finding that **10d** displayed cellular cytotoxicity in human MT-4 cells at >7 μM, which was evaluated within the range of its inhibition concentration, predict that this compound can have desirable drug-like properties, thus making it suitable for further development.

## Figures and Tables

**Table 1:** HIV-1 RT-associated RNase H, RT-associated DNA polymerase, and HIV-1 IN inhibition activities for **9a**–**d** and **10a**–**d**, and the previously described inhibitor RDS1759 [14], used as reference compound.

Compound	RNase H IC_50_ (µM) ^a^	DP IC_50_ (µM) ^b^	IN-LEDGF IC_50_ (µM) ^c^
**9a**	>100	ND	ND
**9b**	>100	ND	ND
**9c**	>100	ND	ND
**9d**	63 ± 7	>100	>100
**10a**	>100	ND	ND
**10b**	>100	ND	ND
**10c**	26 ± 3	90 ± 5	>100
**10d**	**6.6 ± 0.5**	**33.4 ± 5.8**	**>100**
**RDS1759**	7.0 ± 1.5	ND	ND

^a^ Compound concentration required to inhibit the HIV-1 RNase H activity by 50%; ^b^ Compound concentration required to inhibit the HIV-1 DP activity by 50%; ^c^ Compound concentration required to inhibit the HIV-1 IN catalytic activity by 50% in the presence of LEDGF. ND, not determined. Inhibition activities of the most active compound **10d** are highlighted in bold.

**Table 2:** Predicted physicochemical/ADME properties for compounds **9a**–**d**, **10a**–**d**, and RDS1759.

Compound	*M*_W_	H-acc	H-don	Rbond	log P (*o*/*w*)	log S	TPSA
**9d**	370.5	4	1	7	3.328	−6.216	91
**10c**	372.3	2	1	3	1.829	−4.885	65
**10d**	442.4	2	1	7	3.928	−7.396	65
RDS1759	359.8	3	2	8	4.466	−4.103	69

Abbreviations: *M*_W_, molecular weight; H-acc, number of hydrogen bond acceptors; H-don, number of hydrogen bond donors; Rbond, number of rotatable bonds; log P (*o*/*w*), log of the octanol-water partition coefficient ; log S, log of the aqueous solubility; TPSA, topological polar surface area.

## Supplementary Materials

Supplementary materials can be found at www.mdpi.com/1422-0067/17/8/1371/s1.

## Abbreviations

RNase H	Ribonuclease H
RT	Reverse Transcriptase
RNA	Ribonucleic Acid
DNA	Deoxyribonucleic Acid
ADME	Absorption Distribution Metabolism Excretion
HIV-1	Human Immunodeficiency Virus Type-1
AIDS	Acquired Immune Deficiency Syndrome
HAART	Highly Active Antiretroviral Therapy
PR	Protease
IN	Integrase
vRNA	Viral RNA
vDNA	Viral DNA
NRTI	Nucleoside Reverse Transcriptase Inhibitor
NtRTI	Nucleotide Reverse Transcriptase Inhibitor
NNRTI	Non-Nucleoside Reverse Transcriptase Inhibitor
HID	2-Hydroxyisoquinoline-1,3(2*H*,4*H*)-dione
CAIs	Carbonic Anhydrase Inhibitors
SAR	Structure-Activity Relationships
CuAAC	Copper-Catalyzed Azide-Alkyne Cycloaddition
IPTG	β-d-1-thio Galactopyranoside
HEPES	4-(2-hydroxyethyl)-1-piperazineethanesulfonic acid
DTT	Dithiothreitol
EDTA	Ethylenediaminetetraacetic Acid
