# Supplementary material for: Inhibitory Effect of 2,3,5,6-Tetrafluoro-4-[4-(aryl)-1H-1,2,3-triazol-1-yl]benzenesulfonamide Derivatives on HIV Reverse Transcriptase Associated RNase H Activities
Source: Int J Mol Sci. 2016 Aug 20;17(8):1371. doi: 10.3390/ijms17081371 (PMC5000766; doi:10.3390/ijms17081371)
Supplement: Supplementary file 1 [file ijms-17-01371-s001.pdf]

Supplementary Materials: Inhibitory Effect of 2,3,5,6-Tetrafluoro-4-[4-(aryl)-1*H*-1,2,3-triazol-1-yl] benzenesulfonamide Derivatives on HIV Reverse Transcriptase Associated RNase H Activities

Nicolino Pala, Francesca Esposito, Dominga Rogolino, Mauro Carcelli, Vanna Sanna, Michele Palomba, Lieve Naesens, Angela Corona, Nicole Grandi, Enzo Tramontano and Mario Sechi

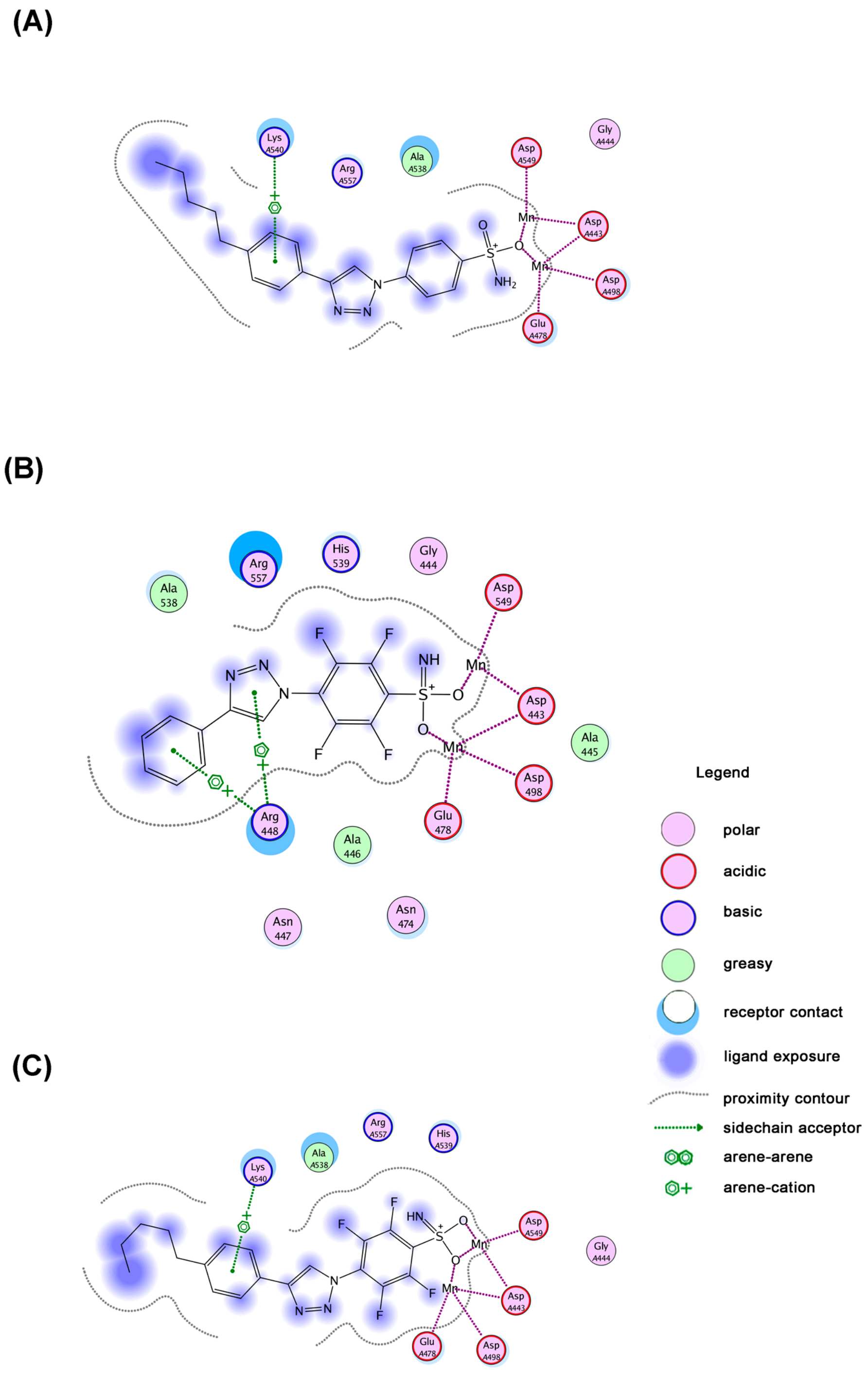

Figure S1. Ligand interaction maps of compounds **9d** (**A**); **10c** (**B**); and **10d** (**C**).

1. Experimental Chemistry: Preparation of Compounds 9a–d and 10a–d (Scheme S1)

1.1. General

All solvents and other chemicals were obtained from Sigma-Aldrich or Carlo Erba (analytical grade and were used without further purification). All reactions involving air- or moisture-sensitive compounds were performed under nitrogen atmosphere using oven-dried glassware and syringes to transfer solutions. Melting points (Mp) were determined using an electrothermal melting point or a Köfler apparatus and are uncorrected. Nuclear magnetic resonance (^{1}H-NMR) spectra were determined in $CDCl_3$ and DMSO-d_6 on a Varian XL-200 (200 MHz) spectrometer. Chemical shifts are reported in parts per million (ppm) downfield from tetramethylsilane (TMS), used as an internal standard. Splitting patterns are designated as follows: s, singlet; d, doublet; t, triplet; q, quadruplet; m, multiplet; brs, broad singlet; dd, double doublet. The assignment of exchangeable protons (O*H* and N*H*) was confirmed by the addition of D_2O. Electron ionization mass spectra were recorded on a Hewlett-Packard 5989 Mass Engine Spectrometer. Analytical thin-layer chromatography (TLC) was carried out on Merck silica gel F-254 plates. Flash chromatography purifications were performed on Merck Silica gel 60 (230–400 mesh ASTM) as a stationary phase. Elemental analyses for tested compounds were performed on a Perkin-Elmer 2400 spectrometer at Laboratorio di Microanalisi, Dipartimento di Chimica, Università di Sassari (Italy), and were within ±0.4% of the theoretical values (Table S1).

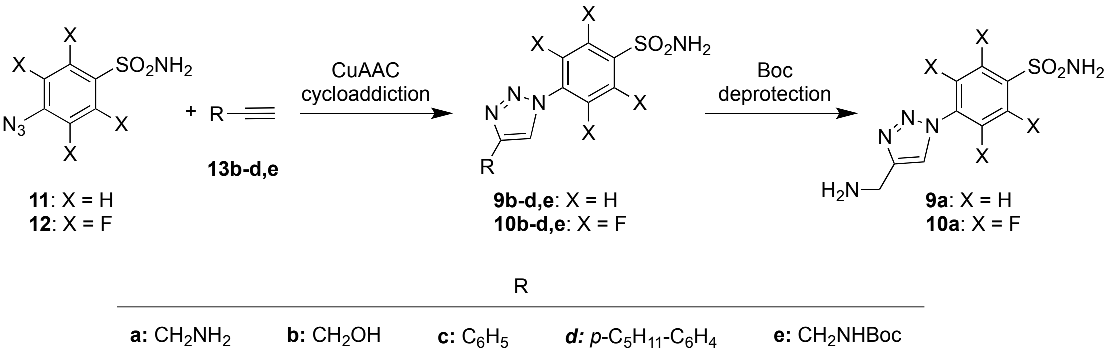

Scheme S1. Synthetic route for the preparation of compounds **9a**–**d** and **10a**–**d**.

1.2. Experimental Methods

Compounds **11** and **12** were prepared according to the previous reported procedures [28].

*1.3. General Procedure for the Preparation of Compounds **9a**–**d**, and **10a**–**d***

To a stirred solution of the appropriate azide (**11** or **12**, 1.0 mmol) in a 1:1 solution of *tert*-butanol and water, alkyne (**13b**–**d**,**e** 1.5 mmol, 1.5 equiv), nanosized activated powder Cu(0) (0.1 mmol, 0.1 equiv), and triethylamine hydrochloride (1.0 mmol, 1.0 equiv) were added in sequence. After stirring at room temperature for 20–28 h, the reaction was complete (as monitored by TLC). The reaction mixture was then diluted with water, extracted with dichloromethane (five times), and dried with anhydrous sodium sulfate. After removal of solvent under vacuum the crude product was treated depending on the case.

4-(4-(Aminomethyl)-1H-1,2,3-triazol-1-yl)benzenesulfonamide hydrochloride (**9a**). The *tert*-butyl 1-(4-sulfamoylphenyl)-1*H*-1,2,3-triazol-4-ylcarbamate (**9e**) was deprotected in an ice-cooled one-neck balloon by treatment with 4 M hydrochloric acid in dioxane (25 mL). The reaction mixture was stirred under nitrogen atmosphere for 90 min and then evaporated to obtain a beige paste. The product was triturated with diethyl ether and filtered to furnish a white powder in 70% yield. Mp 225 °C; ^{1}H-NMR-200 MHz (DMSO-d_6): δ 8.93 (s, 1H, Ar–H); 8.49 (brs, 2H, NH_2, exchange with D_2O);

8.20–8.10 (m, 4H, Ar–H); 7.50 (brs, 2H, SO_2NH_2, slight exchange with D_2O); 4.24 (s, 2H, CH_2); ESI: *m*/*z* 254 [M + 1]$^+$.

4-(4-(Hydroxymethyl)-1H-1,2,3-triazol-1-yl)benzenesulfonamide (**9b**). The crude product was purified by flash chromatography (eluents petroleum ether/ethyl acetate = 3:7) to obtain a grey powder in 55% yield. Mp 195–197 °C; ^{1}H-NMR-200 MHz (DMSO-*d*$_6$): δ 8.80 (s, 1H, Ar–H); 8.18–8.00 (m, 4H, Ar–H); 7.50 (brs, 2H, SO_2NH_2, exchange with D_2O); 5.40 (t, 1H, OH); 4.60 (d, 2H, CH_2); ESI: *m*/*z* 255 [M + 1]$^+$.

4-(4-Phenyl-1H-1,2,3-triazol-1-yl)benzenesulfonamide (**9c**). After purification by flash chromatography (eluents petroleum ether/ethyl acetate = 5:5) a green solid in 29% yield was obtained. Mp 260–261 °C; ^{1}H-NMR-200 MHz (DMSO-*d*$_6$): δ 9.43 (s, 1H, Ar–H); 8.18 (d, 2H, Ar–H); 8.05 (d, 2H, Ar–H); 7.93–7.78 (m, 2H, Ar–H); 7.52 (brs, 2H, SO_2NH_2, exchange with D_2O); 7.55–7.40 (m, 3H, Ar–H); ESI: *m*/*z* 301 [M + 1]$^+$.

4-(4-(4-Pentylphenyl)-1H-1,2,3-triazol-1-yl)benzenesulfonamide (**9d**). After purification by flash chromatography (eluents petroleum ether/ethyl acetate = 7:3) the crude product was triturated with petroleum ether to obtain the title compound as green solid in 24% yield. Mp 253–256 °C; ^{1}H-NMR-200 MHz (DMSO-*d*$_6$): δ 9.39 (s, 1H, Ar-H); 8.19 (d, 2H, Ar-H); 8.05 (d, 2H, Ar-H); 7.87 (d, 2H, Ar-H); 7.53 (brs, 2H, SO_2NH_2, exchange with D_2O); 7.34 (d, 2H, Ar-H); 2.63 (t, 2H, CH_2); 1.61 (m, 2H, CH_2); 1.31 (m, 5H, CH_2, CH_3); 0.88 (m, 2H, CH_2); ESI: *m*/*z* 371 [M + 1]$^+$.

4-(4-(Aminomethyl)-1H-1,2,3-triazol-1-yl)-2,3,5,6-tetrafluorobenzenesulfonamide (**10a**). After purification by flash chromatography (eluents petroleum ether/ethyl acetate = 7:3), *tert*-butyl (1-(2,3,5,6-tetrafluoro-4-sulfamoylphenyl)-1*H*-1,2,3-triazol-4-yl)methylcarbamate (**10e**) was deprotected in a ice-bath cooled round-bottomed flask, by treatment with 4 M HCl in dioxane (20 mL). The reaction mixture was stirred in anhydrous conditions for 2 h. Then the solvent was removed under vacuum and the residue was triturated with petroleum ether/diethyl ether to obtain a white solid in 41% yield. m.p. 231–232 °C; ^{1}H-NMR-200 MHz (DMSO-*d*$_6$): δ 8.73 (brs, 2H, NH_2, exchange with D_2O); 8.70 (s, 1H, Ar-H); 8.58 (brs, 2H, SO_2NH_2, exchange with D_2O); 4.28 (s, 2H, CH_2); ESI: *m*/*z* 326 [M + 1]$^+$.

2,3,5,6-Tertrafluoro-4-(4-(hydroxymethyl)-1H-1,2,3-triazol-1-yl)benzenesulfonamide (**10b**). Purification by flash chromatography (eluents petroleum ether/ethyl acetate = 4:6) followed by trituration with petroleum ether furnished the title compound as white powder (yield 45%). Mp 133–134 °C; ^{1}H-NMR-200 MHz (DMSO-*d*$_6$): δ 8.60 (brs, 2H, SO_2NH_2, exchange with D_2O); 8.50 (s, 1H, Ar-H); 5.49 (brs, 1H, OH, exchange with D_2O); 4.66 (s, 2H, CH_2); ESI: *m*/*z* 327 [M + 1]$^+$.

2,3,5,6-Tetrafluoro-4-(4-phenyl-1H-1,2,3-triazol-1-yl)benzenesulfonamide (**10c**). The crude product was purified by flash chromatography (petroleum ether/ethyl acetate = 7:3) to obtain a yellow solid in 60% yield. Mp 227–228 °C; ^{1}H-NMR-200 MHz (DMSO-*d*$_6$): δ 9.15 (s, 1H, Ar–H); 8.59 (brs, 2H, SO_2NH_2, exchange with D_2O); 7.96 (m, 2H, Ar–H); 7.60–7.44 (m, 3H, Ar-H); ESI: *m*/*z* 373 [M + 1]$^+$.

2,3,5,6-Tetrafluoro-4-(4-(4-pentylphenyl)-1H-1,2,3-triazol-1-yl)benzenesulfonamide (**10d**). The crude residue was purified by flash chromatography (eluents petroleum ether/ethyl acetate = 8:2) to give a grey paste that was triturated with petroleum ether to furnish a white powder (52% yield). Mp 197–198 °C; ^{1}H-NMR-200 MHz (DMSO-*d*$_6$): δ 9.04 (s, 1H, Ar–H); 8.54 (brs, 2H, SO_2NH_2, exchange with D_2O); 7.85 (d, 2H, Ar–H); 7.33 (d, 2H, Ar–H); 2.63 (t, 2H, CH_2); 1.61 (m, 2H, CH_2); 1.30 (m, 5H, CH_2, CH_3); 0.85 (m, 2H, CH_2); ESI: *m*/*z* 443 [M + 1]$^+$.

Table S1. Elemental analysis data of title compounds.

Compound	Molecular Formula	Calculated			Found		
		% C	% H	% N	% C	% H	% N
**9a**	$C_9H_{11}N_5O_2S{\cdot}HCl$	37.31	4.17	24.17	37.22	4.34	24.10
**9b**	$C_9H_{10}N_4O_3S$	42.51	3.96	22.03	42.45	4.10	21.75
**9c**	$C_{14}H_{12}N_4O_2S$	55.99	4.03	18.65	55.96	4.12	18.69
**9d**	$C_{19}H_{22}N_4O_2S$	61.60	5.99	15.12	61.70	6.13	15.02
**10a**	$C_9H_7F_4N_5O_2S{\cdot}HCl$	29.89	2.23	19.36	29.64	2.30	18.47
**10b**	$C_9H_6F_4N_4O_3S$	33.14	1.85	17.17	32.97	1.85	17.01
**10c**	$C_{14}H_8F_4N_4O_2S$	45.17	2.17	15.05	45.11	2.46	14.73
**10d**	$C_{19}H_{18}F_4N_4O_2S$	51.58	4.10	12.66	51.61	4.18	12.43
